# In *Candida parapsilosis* the *ATC1* Gene Encodes for an Acid Trehalase Involved in Trehalose Hydrolysis, Stress Resistance and Virulence

**DOI:** 10.1371/journal.pone.0099113

**Published:** 2014-06-12

**Authors:** Ruth Sánchez-Fresneda, María Martínez-Esparza, Sergi Maicas, Juan-Carlos Argüelles, Eulogio Valentín

**Affiliations:** 1 Departamento de Genética y Microbiología, Facultad de Biología, Universidad de Murcia, Campus de Espinardo, Murcia, Spain; 2 Departamento de Bioquímica, Biología Molecular (B) e Inmunología, Facultad de Medicina, and Regional Campus of International Excellence “Campus Mare Nostrum", Universidad de Murcia, Campus de Espinardo, Murcia, Spain; 3 Departamento de Microbiología y Ecología, Facultad de Farmacia, Universidad de Valencia, Burjassot, Valencia, Spain; 4 Departamento de Microbiología y Ecología, Facultad de Biología, Universidad de Valencia, Burjassot, Valencia, Spain; Newcastle University, United Kingdom

## Abstract

An ORF named *CPAR2-208980* on contig 005809 was identified by screening a *Candida parapsilosis* genome data base. Its 67% identity with the acid trehalase sequence from *C. albicans* (*ATC1*) led us to designate it *CpATC1*. Homozygous mutants that lack acid trehalase activity were constructed by gene disruption at the two *CpATC1* chromosomal alleles. Phenotypic characterization showed that *atc1Δ* null cells were unable to grow on exogenous trehalose as carbon source, and also displayed higher resistance to environmental challenges, such as saline exposure (1.2 M NaCl), heat shock (42°C) and both mild and severe oxidative stress (5 and 50 mM H_2_O_2_). Significant amounts of intracellular trehalose were specifically stored in response to the thermal upshift in both wild type and mutant strains. Analysis of their antioxidant activities revealed that catalase was only triggered in response to heat shock in *atc1Δ* cells, whereas glutathione reductase was activated upon mild oxidative stress in wild type and reintegrant strains, and in response to the whole set of stress treatments in the homozygous mutant. Furthermore, yeast cells with double *CpATC1* deletion were significantly attenuated in non-mammalian infection models, suggesting that *CpATC1* is required for the pathobiology of the fungus. Our results demonstrate the involvement of *Cp*Atc1 protein in the physiological hydrolysis of external trehalose in *C. parapsilosis*, where it also plays a major role in stress resistance and virulence.

## Introduction

Several yeast species are included among the most dangerous microorganisms that cause opportunistic infections in humans and mammals. The genus *Candida,* particularly *Candida albicans,* remains the most prevalent etiological agent of systemic mycoses, but since the 1980s several clinical surveys [1], [2] have documented the increasing impact of “non-*C. albicans”* outbreaks in the bloodstream, e.g. *C. glabrata* in the USA and *C. parapsilosis* and *C. tropicalis* in Europe, Canada and Latin America [3]. Although often considered less virulent than *C. albicans, C. parapsilosis* is the *Candida* species with the largest increase in clinical incidence in recent decades [4], [5]. It causes multifaceted pathologies in immunocompromised and normal hosts, especially low birth weight neonates. The pathological emergence of *C. parapsilosis* may be related to its great ability to colonize the skin, proliferate in sugar-containing solutions, and adhere to plastic-made clinical tools and devices [6].

The dramatic extension of opportunistic mycosis, especially among the debilitated and ageing population, and the worrying isolation of fungal strains resistant to conventional antibiotics points to the need for more efficient and selective antifungal compounds. In this context, the non-reducing disaccharide trehalose has been studied as a potentially interesting antifungal target [7]–[9]. Trehalose (alpha-D-glucopyranosyl (1–1) alpha-D-glucopyranoside) is widely present in many organisms including yeasts, fungi, bacteria, plants and insects, but not in mammals [10], [11]. The synthesis of intracellular trehalose plays important functions in yeasts. It constitutes an endogenous storage of carbon and energy, it acts as stabilizer of cellular membranes and proteins and also functions as stress protector in yeast and fungi [10]–[12]. Trehalose synthesis takes place in a sequential two-step reaction: trehalose 6-phosphate is synthesized from UDP-glucose and glucose 6-phosphate in a reaction catalyzed by a Mg-dependent trehalose 6-phosphate synthase (coded by the *TPS1* gene). Then, a trehalose phosphatase, coded by the gene *TPS2*, dephosphorylates trehalose-6-phosphate to release free trehalose. Trehalose hydrolysis is essentially confined to a specific class of α-glucosidases that cleaves off the disaccharide, rendering two molecules of glucose, the enzyme trehalase (E.C.3.2.1.28) [10]–[12]. Most fungi possess two specialized and apparently unrelated trehalases, which differ in location, catalytic properties and regulation. The neutral trehalase is a cytosolic enzyme with maximal activity at neutral pH (7.0), activated by Ca^2+^ or Mn^2+^ and regulated by cAMP-dependent protein kinases. For its part, the so-called acid trehalase (optimum pH about 4.5) is located inside the vacuoles (*Saccharomyces cerevisiae*) or is associated to the cell wall (*Candida albicans*), whose activity is subjected to glucose repression [13]. We and others have previously demonstrated that the enzymes involved in the trehalose biosynthetic and hydrolytic pathways act as virulence factors in *C. albicans* [7], [14]–[17]. To our knowledge, there is no information available concerning trehalose metabolism in the emergent opportunistic pathogen *C. parapsilosis*. In this work, we describe the cloning of the *CpATC1* gene, which encodes an acid trehalase homologous to *C. albicans* Atc1 [13]. The role of *CpATC1* was examined in response to different *in vitro* stress challenges and in the new safer model of infectivity *Galleria melonella* [18]. Our results support the view that the homozygous *atc1*Δ null mutant is unable to grow on trehalose as carbon source, confirming that *Cp*Atc1 activity is required to hydrolyze exogenous trehalose. The *CpATC1*-deficient mutants were more resistant to *in vitro* stress exposure, but more sensitive to immune system clearance after *in vivo* infection than its parental counterpart, suggesting a major role for the *CpATC1* gene in *C. parapsilosis* virulence.

## Material and Methods

### Ethics statement

All experimental procedures were approved by the local Ethical Committee for Animal Experimentation of the University of Murcia (CEEA-UM).

### In silico analysis of CpATC1

The presence of an N-terminal signal peptide was analyzed using SignalP (http://www.cbs.dtu.dk/services/SignalP/). The Kyte-Doolittle hydropathy plot was generated using a webserver at the University of Virginia (http://fasta.bioch.virginia.edu/fasta_www2/fasta_www.cgi?rm=misc1). For pattern scanning we used ProFASTA (http://www.bioinformatics.nl/tools/profasta/) [19] and for homology searching, NCBI-Blast (http://blast.ncbi.nlm.nih.gov/Blast.cgi).

The predicted amino acid sequence (residues 1-1039 of the mature protein) of the *Cp*Atc1 protein was submitted to the JIGSAW 3D Protein Homology Modelling Server [20], which split the protein into two domains, both with successful structural template matching the catalytic domain of glycosidases. The sequence was also submitted to the Phyre2 Server to generate an accurate homology model [21]. Two domains, the catalytic domain of glycosidases and a carbohydrate-binding domain were recognized. Structural alignments were refined by visual inspection and using the secondary-structure (SSM) server [22]. A superimposed global model was generated involving almost the complete sequence. The entire sequence was also submitted to the 3DLigandSite [23] to predict the protein binding site.

### Strains and Growth Conditions

The *C. parapsilosis* strains used or originated during this study are listed in Table 1. The cells were grown in YP medium (1% yeast extract, 2% peptone) or MM medium (0.7% yeast nitrogen base without amino acids and ammonium sulfate) supplemented with 0.5% ammonium sulfate and appropriate nutrients, as detailed in [24], and with the appropriate carbon source (2% glucose, 2% maltose or 2% trehalose). Media were solidified with 2% agar. *C. parapsilosis* transformed isolates were grown in YPD (2% glucose) containing 200 µg/ml of nourseothricin (Nou) (Jena Biosciences, Jena, Germany). Nou resistant (Nou^R^) colonies were grown for 24 h in YPM medium. After incubation at 30°C, 200 cells were plated on YPD containing 20 µg/ml of Nou. Nou-sensitive (Nou^S^) colonies were picked up and used for the second round of transformation. *Escherichia coli* DH5α (*F, φ80, lac4M15, recA1, endA1, gyrA96, thi-1, (rK-, mK-), supE44, relA1, deoR, Δ(lacZYA-argF)U169*) strain was grown routinely in LB medium (0.5% yeast extract, 1% tryptone, 0.5% NaCl) supplemented with 100 µg/ml ampicillin or 35 µg/ml cloramphenicol. *E. coli* was transformed as described elsewhere [25].

**Table 1:** *C. parapsilosis* strains constructed and used during this study.

Name	Genotype	Reference
AM2001/0013 Wild type (WT)	*ATC1/ATC1*	Odds, 2008
Atc1 HET^R^ (Nou^R^)	*ATC1*/*Δatc1::SAT1-FLP*	This study
Atc1 HET (Nou^S^)	*ATC1*/*Δatc1::FRT*	This study
Atc1 KO^R^ (Nou^R^)	*Δatc1::FRT/Δatc1::SAT1-FLP*	This study
Atc1 KO (Nou^S^)	*Δatc1::FRT/Δatc1::FRT*	This study
Atc1 RE (Nou^S^)	*ATC1*/*Δatc1::FRT*	This study

R Resistant; ^S^Sensitive.

### Plasmid Construction

To generate acid trehalase-negative mutants, the *SAT1*-flipper method [26] was used. Plasmid pCD8 was kindly provided as a gift by Dr G. Butler, University College Dublin, Ireland. It contains the *C. albicans* Nou resistance gene under the control of a *C. parapsilosis* actine promoter (*ACT1*) and a recombinase *FLP* gene whose expression is driven from the *C. parapsilosis* maltose promoter *(MAL2)* [27].

To disrupt *CpATC1* gene, a 621 bp fragment was amplified from the upstream region using primers F*Cp*ATC1-5 (5′-AAACTTGGTACCTCGTGGATGGTTATTTTCTCTTCC-3′) and R*Cp*ATC1-5 (5′-AAACTTGGGCCCATCTCCTAATACCTTTGATTCTGG-3′) containing engineered *Kpn*I and *Apa*I restriction sites, and a 495 bp fragment was amplified from the downstream region using primers F*Cp*ATC1-3 (5′-AAACTTCCGCGGATTAGAGCCCAAAAGCAATAAC-3′) and R*Cp*ATC1-3 (5′-AAACTTGAGCTCTGAATGAGCAACCACCAGCGGC-3′) containing engineered *Sac*II and *Sac*I restriction sites respectively (underlined). Those amplicons were introduced into pCD8, generating plasmid pRES12. A fragment excised with *KpnI* and *Sac*I from pRES12 was used for *C. parapsilosis* transformation.

In order to rescue the acid trehalase activity in the KO strain, the vector pRESR was constructed. Using primers F*Cp*ATC1-5 and R*Cp*ATC1-3, and a proofreading DNA polymerase (Expand High Fidelity^PLUS^, Roche, Barcelona, Spain) an amplicon of 3216 bp was obtained, cloned into the vector pJET1.2/blunt (Fermentas, Ottawa, Canada) and verified by sequencing. The plasmid pRESR was digested with *Kpn*I and *Sac*I and the fragment corresponding to *CpATC1* was blunt-ended and used to transform *C. parapsilosis* KO strain. Transformed cells were selected in MM medium containing trehalose as sole carbon source and checked for correct integration by PCR using the forward primer F*Cp*ATC1-5 and the reverse primer R*Cp*ATC1-55 (5′-TTCAATGTGGTCCATTGTGG-3′) which generated an amplicon of 1.2 kb only if integration had been performed in the correct locus.

### Transformation of *C. parapsilosis*

*C. parapsilosis* strains were transformed by electroporation as described previously by [26] for *C. albicans* with slights modifications. *C. parapsilosis* yeast cells were grown overnight at 30°C in YPD medium and then diluted in 100 ml of fresh YPD and grow to reach an OD_600nm_ =  1.4–2.0. The cells were centrifuged at 3500 xg for 10 min and washed two times with 50 ml of ice-cold water, resuspended in 20 ml of TE (10 mM Tris-HCl, 1 mM EDTA, pH 7.5)/100 mM lithium acetate, pH 7.5, and incubated in a rotary shaker at 150 rpm for 45 min at 30°C. After addition of 500 µl of 1 M dithiotreitol the cells were shaken for an additional 15 min. After addition of 80 ml of ice-cold water, the cells were centrifuged, washed twice with 50 ml water and then with 10 ml 1 M sorbitol and kept on ice. Approximately 2 µg of purified *Kpn*I-*Sac*I from pRES12 or pRESR was mixed with 40 µl of *C. parapsilosis* competent cells and transferred into a 0.2 cm electroporation cuvette. The electroporation was performed at 1.8 kV using a Bio-Rad MicroPulser ™ electroporator. After electroporation cells were washed once in 1 ml of 1 M sorbitol, suspended in 1 ml of YPD medium and incubated at 30°C for 4 h with shaking. Cells were concentrated in 100 µl of YPD and plated onto YPD plates containing 200 µg/ml Nou and grown at 30°C during 2–3 days.

### Southern Blot

Genomic DNA isolation, gel electrophoresis and hybridization was performed as described [28]. Approximately 15 µg of genomic DNA isolated as previously described [29] from *C. parapsilosis* strains were digested with *Bam*HI, separated on a 1% agarose gel and transferred onto a nylon membrane (Roche, Barcelona, Spain). For hybridization, a 621-bp probe obtained with F*Cp*ATC1-5 and R*Cp*ATC1-5 primers (amplicon F1) was labeled by random primed incorporation of a digoxigenin-labeled deoxyuridine triphosphate using the DIG-DNA labeling kit (Roche, Barcelona, Spain) according to the manufacturer's instructions. DNA concentrations were determined by measuring absorbance (A_260_) in a Gene Quant II RNA/DNA calculator spectrophotometer (Amersham Biosciences, Quebec, Canada).

### Stress Treatments

Cultures were grown in YPD until they reached exponential phase (OD_600nm_ = 0.8–1.0) and were then divided into several identical aliquots, which were treated with different H_2_O_2_ concentrations (5–50 mM), 1.2 M NaCl or 42°C for oxidative, osmotic or heat-shock stress treatments, respectively, or maintained without treatment as a control and incubated at 30°C for 1 h. Viability was determined after samples had been diluted appropriately with sterile water by plating in triplicate on solid YPD after incubation for 2–3 days at 30°C. Between 30 and 300 colonies were counted per plate. Survival was normalized to control samples (100% viability). The susceptibility to compounds that interfere with the cell-wall architecture was tested in solid media. Cells were diluted in YPD and 10^5^ cells, and ten-fold dilutions thereof, were spotted in 5 µl onto YPD agar containing the specific compound at the indicated concentration. Plates were incubated at 30°C and scored after 48 h.

### Preparation of cell free extracts and Enzymatic Assays

Cell-free extracts were obtained as described previously by [17], with slight modifications. The yeast cultures were harvested and resuspended at known densities (10–15 mg/ml, wet weight) in 10 mM 2-(*N*-morpholine) ethanesulfonic acid (MES), pH 6.0, containing 1 mM phenylmethylsulfonyl fluoride (PMSF). The cellular suspensions were transferred into small, Eppendorf tubes with 1.5 g Ballotini glass beads (0.45 mm diameter). Cells were broken by vibrating the tubes vigorously in a vortex mixer for 5 min at 4°C. The tubes were then cooled quickly on ice. The crude extract was centrifuged at 10 000 xg for 5 min and the pellet was resuspended in the same buffer at the initial density. Acid trehalase activity was measured as described elsewhere [13]; specific activity was expressed as nmol of glucose released min^−1^ (mg of protein)^−1^. Catalase activity was determined at 240 nm by monitoring the removal of H_2_O_2_ as described previously for *C. albicans* [30]. Glutathione reductase (GR) activity was assayed by measuring the glutathione disulfide (GSSG)-dependent oxidation of NADPH as described elsewhere [31].

### *Galleria mellonella* Survival Assay

*G. mellonella* larvae (R. J. Mous Livebait, The Netherlands) were infected as described previously [32]. Groups of 20 larvae (0.3–0.6 g) were inoculated with 10 µl of 2.5×10^6^ yeast cells/ml in PBS supplemented with ampicillin (20 µg/ml) to avoid bacterial contamination. The yeast cells were directly instilled into the haemocele of the larvae by injection using a 26-gauge needle with Hamilton syringe in the last left proleg. The larvae were incubated at 37°C after inoculation, and survival was monitored every day. Larvae death was assessed by the lack of movement in response to stimulation together with darkening of the cuticle. In parallel, as control, a group of non-infected larvae and a group of larvae inoculated with PBS-ampicillin were studied in every infection. Each experiment was repeated at last three times, and representative experiments are presented.

### *In vivo* Phagocytosis Assay

Yeast cells were stained with 10 µl/ml Calcofluor white (Sigma Aldrich, St. Louis, MO, USA) for 30 min at 37°C in darkness and injected into *G. mellonella* larvae (10^7^ cells/larva, 10 per group). After 4 h of incubation at 37°C, haemolymph was collected in 1.5 ml tubes and diluted 1∶1 in IPS buffer (Insect Physiological saline: 150 mM sodium chloride, 5 mM potassium chloride, 10 mM Tris-HCl pH 6.9, 10 mM EDTA and 30 mM sodium citrate) to avoid coagulation and melanization of the haemolymph. Haemocytes were placed on a slide and phagocytosis was quantified visually using a Leica DMI 3000B fluorescence microscope. One hundred haemocytes from each larva were counted in each case, and the percentage of haemocytes containing yeast was calculated and plotted.

### Murine infection models

Swiss CD-1 female mice, 6–8 weeks of age (Animal facilities of the University of Murcia) weighting 25–30 g each, were inoculated intraperitoneally with 30×10^6^ fungal cells in 150 µl PBS. The mice were sacrificed 3 days after infection. Kidneys were removed and organ homogenates plated on YPD to count the CFUs.

### Statistical Analysis

The statistical analysis was performed using GraphPad Prism version 5.02 for Windows (GraphPad Software, San Diego, California, USA). The significance of differences between sets of data was determined by Student´s t-test. Killing curves were plotted and differences in survival (Log rank and Wilcoxon test) were analyzed by the Kaplan-Meier method. Every experiment was repeated at least twice, and similar results were obtained in all cases.

## Results

### In silico screening for potential acid trehalases in *C. parapsilosis*

**Figure 1:**
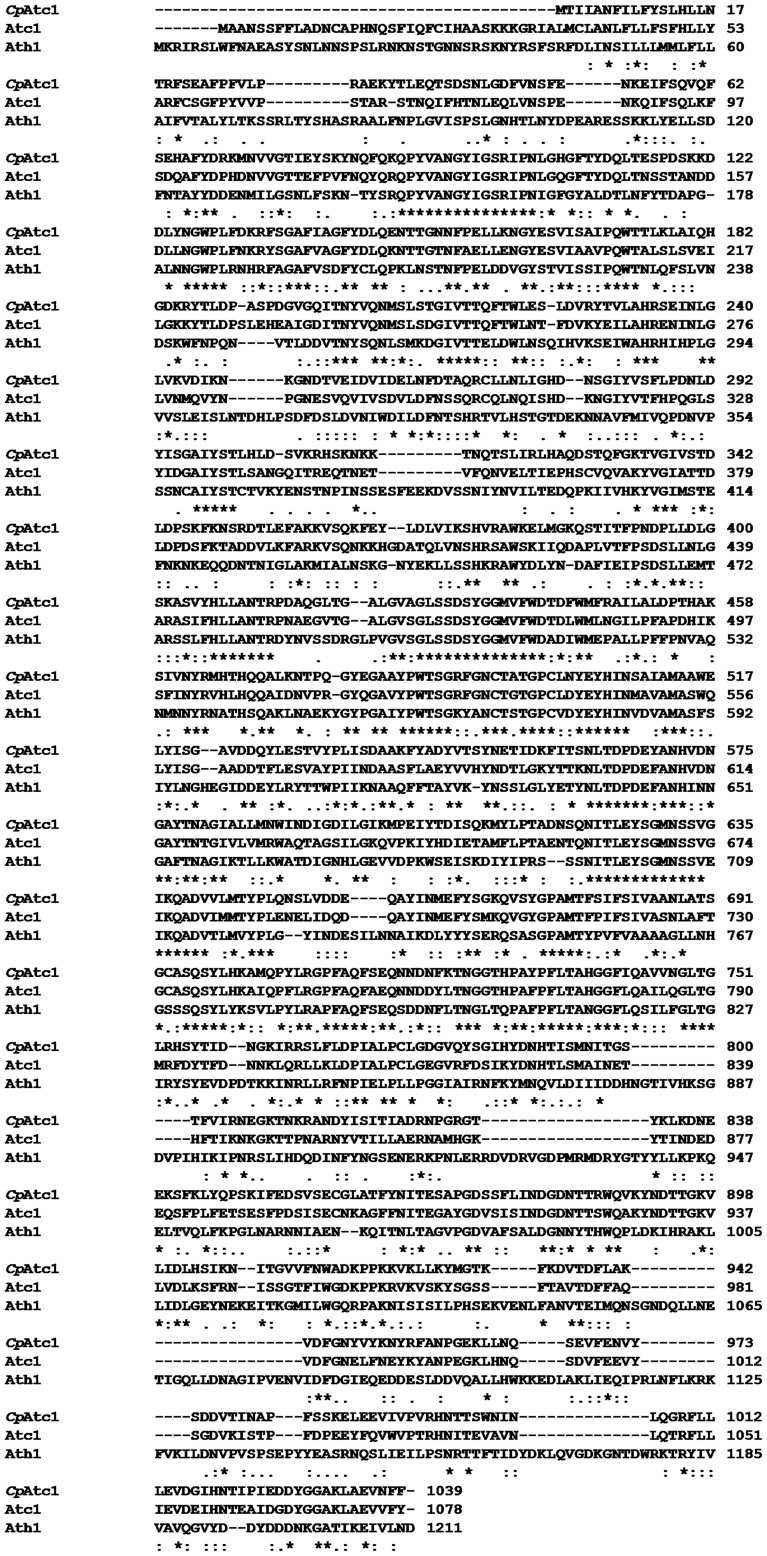
Alignment of *Cp*Atc1 amino acid sequence from *C. parapsilosis* with Atc1 from *C. albicans*, and Ath1 from *S. cerevisiae*. The text indicates the residues that are identical (*), conserved substitutions residues (:) and semi-conserved substitutions (.). Dashes represent gaps to maximize alignment.

*The C. parapsilosis* genome database (www.sanger.ac.uk/resources/downloads/fungi/candida-parapsilosis.html) was blasted taking the gene sequence of the acid trehalase from *C. albicans* (*ATC1*) as template. An ORF named *CPAR2-208980* was found on contig 005809, which presented 67% nucleotide sequence homology with *ATC1* from *C. albicans* [13], and 62% homology at protein level. Similar homology was found after comparison of the predicted amino acid sequence with the sequences found in protein data bases using the BLAST search algorithm [33] for *C. albicans* Atc1 [13] and *S. cerevisiae* Ath1 [34]. In comparisons made over the entire length, *CPAR2-208980* shared 62% identical and 77% similar amino acids with Atc1, and 41% identical and 58% similar amino acids with Ath1 (Figure 1). These *in silico* results suggest that ORF *CPAR2-208980* encodes *C. parapsilosis* acid trehalase, leading us to name the gene *CpATC1*.

### Structural Analysis of the amino acid sequence encoded by *CpATC1*

**Figure 2:**
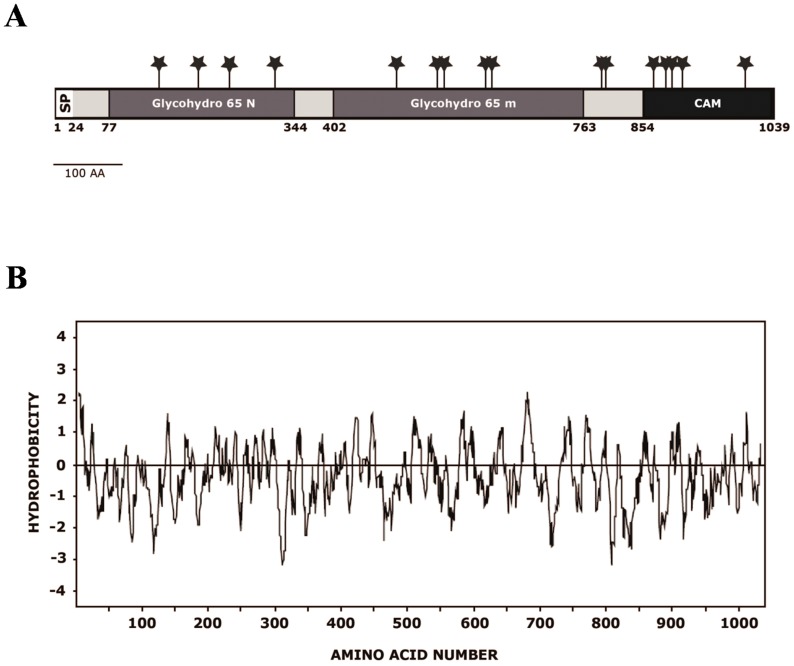
Amino acid sequence of *Cp*Atc1. (**A**) Diagram of the features of *Cp*Atc1. The hydrophobic N-terminal domain corresponding to a signal peptide (amino acids 1-22), the potential N-glycosylation sites (Asn-Xaa-Ser/Thr) (*), the regions showing homology with the glycosyl hydrolase family 65 N-terminal domain (Glycohydro 65N) and with glycosyl hydrolase family 65 central catalytic domain (Glycohydro 65 m), are indicated. (**B**) Hydropathic plot from the deduced amino acid sequence of *Cp*Atc1. Values above and below the horizontal line indicate hydrophobic and hydrophilic regions, respectively.

The ORF *CPAR2-208980* on contig 005809 encodes a putative polypeptide of 1039 amino acids with a calculated molecular weight of 116587.90 D and a pI of 5.45. Analysis of the predicted amino acid sequence revealed an N-terminal region with the characteristics of a signal peptide [35] and a predicted cleavage site between positions 23 and 24 (…SEA-FP) (Figure 2A). Hydropathy analysis [36] of the deduced amino acid sequence showed that the hydrophobic signal sequence is followed by a neutral region representing the mature protein (Figure 2B). Sixteen potential N-glycosylation sites (NXT(S)/T) were identified at amino acid positions 149, 206, 251, 317, 493, 552, 562, 622, 631, 790, 796, 865, 883, 892, 907, 998 (Figure 2A).

A search of protein motifs in *Cp*Atc1 revealed two significant matches with the fungal trehalases family. Located in the N-terminal portion of the 1039 amino acid length protein, there is a glycosyl hydrolase family 65 motif in which amino acids 77–344 are involved. The central segment also correlates with the glycosyl hydrolase 65 family catalytic domain (amino acids 402–763) (Figure 2A). This family, included in the GH-L clan, comprises glycosyl hydrolases (α/α)_6,_ such as vacuolar acid trehalase and maltose phosphorylase, according to the Carbohydrate–Active Enzyme (CAZy) classification (http://www.cazy.org/fam/GH65.html). This last crystalized enzyme (MPLb) catalyzes the conversion of maltose and inorganic phosphate into β-D-glucose-1-phosphate and glucose. The central region corresponds to the catalytic domain, which binds a phosphate ion that is proximal to a highly conserved Glu.

**Figure 3:**
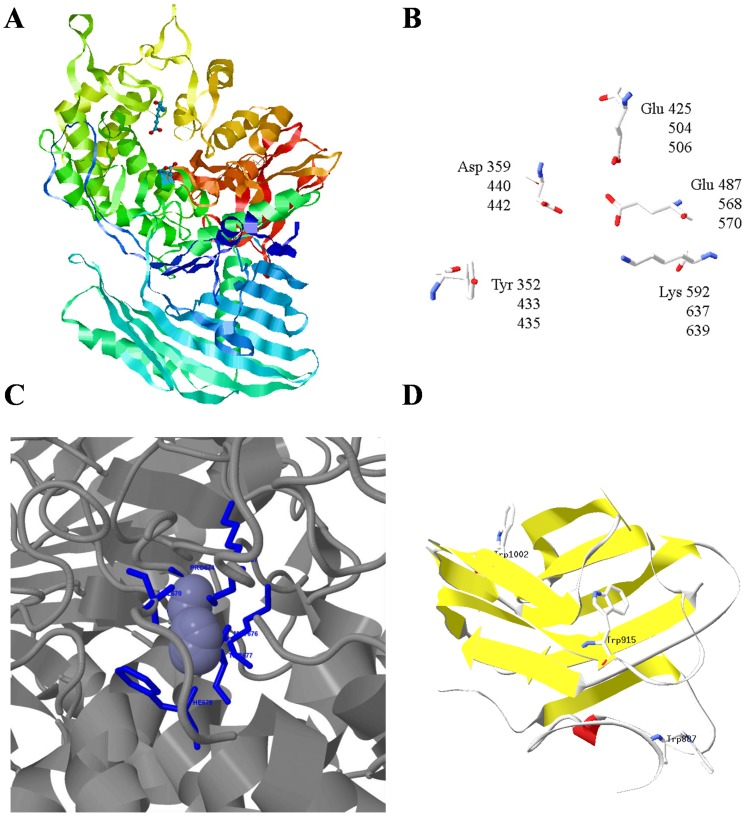
Structural analysis of *Cp*Atc1 protein. (**A**) Ribbon representation. Colour-ramped from the N terminus (blue) to the C terminus (red). The residues proposed to act in the hydrolysis mechanism (Asp440 and Glu568) are shown in ball-and-stick representation. The output structure was generated with RasMol (Sayle and Milner-White, 1995). (**B**) Superimposition of the important active site molecules of *Lactobacillus brevis* maltose phosphorylase pdb:1H54, *C. parapsilosis* and *C. albicans (*Labels are shown in order). The figure represents the best fit between the three molecules. The structure was generated with the Swiss Pdb-viewer (Guex and Peitsch, 1997). (**C**) 3DLigandSite visualization of prediction for the *Cp*Atc1 structure with predicted binding site for Zn (blue). The ligands in the cluster used to make the prediction are displayed with ions in spacefill and organic molecules in wireframe formats. (**D**) Putative homology model for the *Cp*Atc1CBM from *C. parapsilosis* covering residues 854 to 1039. The residues proposed to participate in the binding site (Trp887, Trp915 and Trp1002) are shown in ball-and-stick representation. The structure was generated with the Swiss Pdb-viewer (Guex and Peitsch, 1997).

A satisfactory model was generated by superimposition (estimated precision >90%) comprising the putative catalytic active centre of the enzyme (amino acids 400–600) and the carbohydrate accessory module at the C-terminal region (amino acids 854–1039) (Figure 3A). The maltose phosphorylase enzyme from *Lactobacillus brevis* (pdb entry: 1H54) and the carbohydrate binding module from *Streptococcus pneumoniae* (pdb entry: 2J1R) were automatically used as the best possible available templates in the model generation. The *in silico* analysis of the hypothetical 3-D structure of *Cp*Atc1 protein revealed a (α/α)_6_ toroid folding enzyme, consistent with the six-hairpin glycosidase superfamily (GH-L, GH-M and GH-H clan) [37], [38] classified by the secondary-structure (SSM) server [22]. This overall appearance closely resembled that of CH94 chytobiose phosphorylases [39], GH15 glucoamylases [40] and GH65 maltose phosphorylases [41]. All these enzymes act as catalysts with inversion of the anomeric configuration. The predicted 3-D structure of *Cp*Atc1 offers a satisfactory spatial superimposition of Glu487 (MPLb), Glu570 (Atc1) and Glu568 (*Cp*Atc1) on the one hand and Asp359 (MPLb), Asp442 (Atc1) and Asp440 (*Cp*Atc1) on the other (Figure 3 B). The structural alignment provided by the SSM server confirmed that these crucial amino acids are located in topologically identical loops. Moreover, MPLb Tyr352, Lys592 and Glu425 also coincide with Atc1 Tyr433, Lys637 and Glu504, respectively, which provides side chains that could interact with hydroxyl groups of the substrate. An identical superimposition was corroborated with the *Cp*Atc1 molecule. Glu568 was also surrounded by a cluster of hydrophobic residues that might be considered good candidates for interaction with the trehalose molecule. The automatic submission of our 100% confidence sequence to the 3DLigand site prediction server revealed a binding site for Zn in the central portion of the protein involving Lys637, Val670, Pro674, Met676, THr677 and Phe678 residues (Figure 3 C). In the C-terminal region of the molecule we identified a β-sandwich fold which resembles a sugar-binding domain such as those proposed by [42]. These carbohydrate binding modules (CBMs) have been described as the non-catalytic carbohydrate binding accessory modules from larger enzymes dedicated to the breakdown of polysaccharides. CBMs are believed to be vital for enzyme targeting and substrate concentration [43] and are sometimes involved in substrate presentation for catalysis [44]. The binding site architecture of *Cp*Atc1CBM is a well conserved scaffold comprising variations of an eight-stranded β-sandwich fold (Figure 3 D). A small section of α-helix separates β-strand β1. This is a common fold among carbohydrate binding proteins belonging to the CBM family. The spatial localization of Trp887, Trp915 and Trp1002 in *Cp*Atc1 resembles the binding site of other CBM32 modules [45].

### Disruption of *CpATC1* to generate acid trehalase mutants

**Figure 4:**
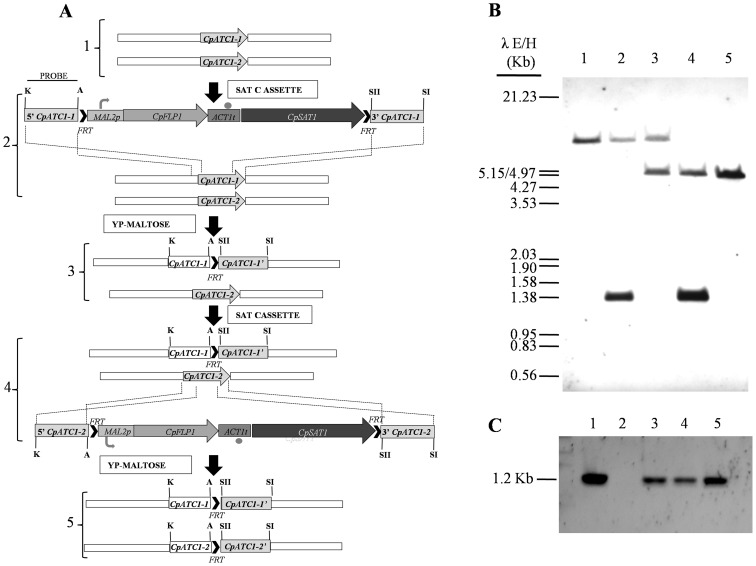
Construction of the *CpATC1* null (KO) and the reintegrant (RE) *C. parapsilosis* strains. (**A**) Diagram of the sequential process followed to disrupt both alleles of *CpATC1* (steps 1–5). The probe used to verify correct integration and deletion of the *SAT1* flipper by Southern blot hybridization is represented by a black line in step 2. (**B**) Southern blot hybridization analysis of genomic DNA digested with *BamH*I and isolated from the wild type of *C. parapsilosis*, (lane 1), HET^R^ (lane 2), HET (lane 3), KO^R^ (lane 4), KO (lane 5). (**C**) The reintegration was confirmed by PCR amplification using the primer pair F*Cp*ATC1-5 and R*Cp*ATC1-4. A positive control (lane 1), a negative control (lane 2) and 3 problem samples (lanes 3–5) are depicted.

The *CpATC1* gene was demonstrated to be essential for *C. albicans* growth on exogenous trehalose as carbon source [13]. To investigate the function of *CpATC1*, homozygous null mutants were constructed by targeted gene disruption, and the resulting phenotypes were analysed. Both alleles of *CpATC1* were disrupted using the *SAT1* flipper cassette originally developed for *C. albicans* [26] and modified by [27] for *C. parapsilosis*. The strategy followed for gene disruption is outlined in Figure 4A. The clinical isolate *C. parapsilosis* (AM2001/0013) strain was transformed by electroporation with a linear DNA fragment, in which 3251 bp containing the ORF were replaced by the insertion of the *SAT1* cassette. The cells treated with electric pulse were incubated at 30°C in liquid YPD containing 1M sorbitol with shaking, prior to plating on YPD containing 200 µg/ml of Nou. After 2 days' incubation at 30°C, the Nou^R^ colonies corresponding to heterozygous mutants (HET^R^), observed on the selection plate could be picked and used to prepare cultures for DNA isolation. The HET^R^ mutants were examined by Southern blot hybridization in order to demonstrate homologous integration. Figure 4B shows the Southern blot with a probe localized outside the homologous regions. Five of the twenty transformants analyzed displayed correct integration of the cassette within the *CpATC1* locus. The HET^R^ homologous integrated heterozygous mutants were inoculated into YP containing maltose as carbon source in order to induce FLP-mediated excision of the *cpSAT1* resistance gene. After 24 h of induction, approximately 200 cells were plated on the YPD plate containing 20 µg/ml Nou. The Nou^S^ cells (HET) grew more slowly and formed smaller colonies compared with the Nou^R^ colonies. Southern blot analysis showed that all of the HET clones lacked the resistance marker (data not shown). One HET colony was used in the second transformation round to inactivate the remaining wild type locus. We analyzed 10 mutants from each transformation and found 3 independent homozygous mutants (KO^R^) that showed the correct integration (Figure 4B). *MAL2* activation and excision of the resistance marker were carried out as described for the first transformation. Finally we obtained the homozygous mutant (KO).

### Construction of *C. parapsilosis CpATC1*-reconstituted strain

To demonstrate that the mutant phenotype was caused by deletion of the *CpATC1* locus, the *CpATC1* gene was reintroduced into the original genomic locus. The first step was to amplify the *CpATC1* gene by PCR, using genomic DNA from the *C. parapsilosis* wild strain as template, and the oligonucleotides F*Cp*ATC1-5 and R*Cp*ATC1-3. The resulting 3216 bp amplicon was sequenced and used to construct plasmid pRESR (see Methods). The homozygous mutants were electroporated with the linear DNA fragment containing the *CpATC1* gene and transformed into the mutant strain lacking the gene. Transformants were selected on YNB plates with trehalose as a carbon source, thereby allowing the exclusive growth of colonies that had incorporated the gene *CpATC1*, which acted in this case as a selection marker. After transformation, three colonies were analyzed by PCR using the primers F*Cp*ATC1-5 and R*Cp*ATC1-4 (5′-TTCAATGTGGTCCATTGTGG-3′), which only amplified an internal sequence of the *CpATC1* gene of 1.2 kb if the integration had occurred at the correct locus (Figure 4C).

### Phenotypic analysis of the *C. parapsilosis CpATC1* mutants

**Figure 5:**
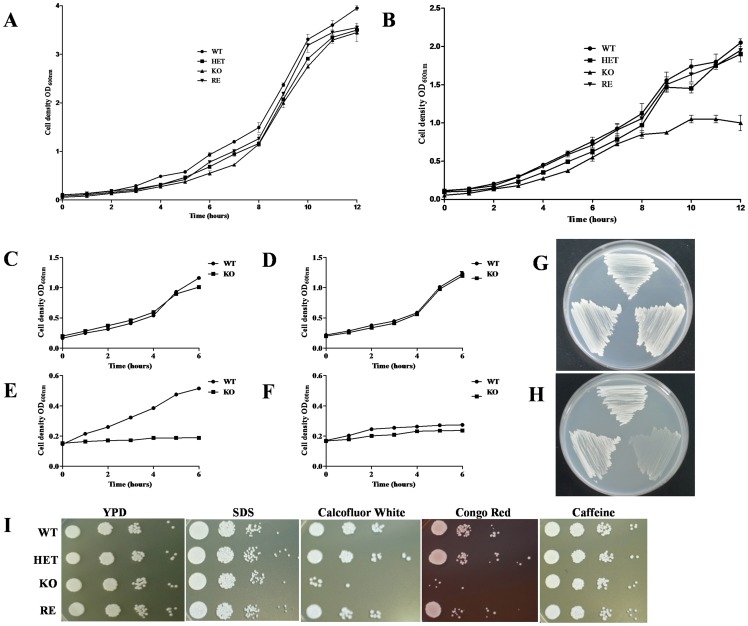
Phenotypic analysis of the *C. parapsilosis* strains. The growth cycle at 30°C of wild type (WT), heterozygous (HET) and homozygous (KO) and reintegrant (RE) yeast strains was monitored in YPD (**A**) or YPtrehalose (**B**). To check the ability to use different carbon sources, cells from the WT and KO strains were cultured in liquid minimal medium (MM) supplemented with: glucose (**C**), sucrose (**D**), trehalose (**E**) and lactose (**F**) at 30°C for 6 hours; or in solid MM medium supplemented with glucose (**G**) or trehalose (**H**) for 24h. The susceptibility to compounds that affect the cell wall architecture (**I**) was examined by spotting approximately 10^5^ cells and 10-fold dilutions thereof, on YPD plates containing the indicated compounds at the following concentrations: SDS (0.02%, w/v); Calcofluor White (60 µg/ml); Congo Red (100 µg/ml) and Caffeine (50 mM). The plates were incubated at 30°C for 48 h and photographed. Growth in liquid medium was measured by cell density at OD_600_. Results are expressed as mean ± standard deviation of one representative experiment of two performed in triplicate. Growth in solid medium was monitored by visual inspection of plates performed in duplicate and repeated twice with similar results.

The growth cycle at 30°C of the *C. parapsilosis* strains used in this study was analyzed in different conditions. The four cell types exhibited a roughly similar growth pattern in YPD rich medium (Fig. 5 A), whereas substitution of glucose by trehalose as carbon source (YPtre medium) caused a certain delay in KO cells after 8 hours of incubation (Fig. 5B). These results suggest the *CpATC1-*deficient mutant might be unable to metabolize exogenous trehalose, the initial growth being sustained by the nutritional ingredients, yeast extract and peptone. To check this hypothesis, only the wild type and the KO null mutant were cultured in liquid minimal medium (MM) supplemented with different carbon sources (glucose, sucrose, trehalose and lactose) (Figure 5 C-F). The results clearly showed that *CpATC1* deletion in *C. parapsilosis* impaired the ability to use trehalose as sole carbon source (Figure 5 E). This yeast was also unable to grow in lactose, as can be seen in Figure 5F and has been previously observed [46]. In addition to the experiments in liquid medium, the growth pattern was also analyzed on solid medium in order to confirm these phenotypes. No significant differences were found when the studied strains were grown on plates of MM-glucose (Figure 5 G), whereas the *CpATC1* homozygous null mutant has lost the capacity to grow on MM plus trehalose as sole carbon source (Figure 5 H). Therefore, these data strongly support the idea that the acid trehalase activity in *C. parapsilosis* is necessary to hydrolyze exogenous trehalose. The phenotypic analysis of the distinct *CpATC1* constructions was completed by measuring the sensitivity to a set of compounds that interfere with the cell wall integrity (Calcofluor White, Congo red, SDS and Caffeine). The *Cp*Atc1 null mutant (KO strain) showed increased sensitivity to Calcofluor White and Congo Red compared to the WT strain (Figure 5 I). The susceptibility to both compounds was restored by reintroduction of a functional *CpATC1* gene (RE strain) (Figure 5 I). In identical drop tests, all the strains displayed a similar degree of susceptibility to SDS and Caffeine (Figure 5 I). These results suggest that disruption of the *CpATC1* gene altered the cell wall structure, which made the cells more sensitive to agents that perturb the cell walls but not to those affecting the cell membranes. However, the evidence is not conclusive and the location of *Cp*Atc1 in the cell wall cannot be unequivocally established, as it can in the case of *C. albicans* [13].

### Level of cellular viability after several stress treatments

**Figure 6:**
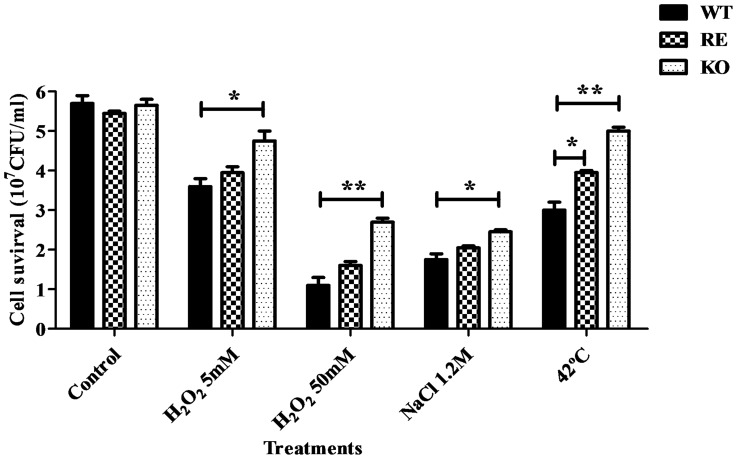
Level of cell survival after different stress treatments in *C. parapsilosis* strains. YPD-grown cultures of exponential *C. parapsilosis* wild type (WT), its isogenic mutant (KO), deficient in *CpATC1* gene, and the reintegrant (RE) strains were adjusted to a cell density of 1.0×10^6^ cells/ml and subjected to the following stress challenges for 1h: 5 mM H_2_0_2_, 50 mM H_2_0_2_, 1.2 M NaCl or 42°C. Identical, untreated samples were maintained at 30°C as a control. Results are expressed as mean ± standard deviation of one representative experiment of two performed in triplicate. Student t-test: *P<0.05; **P<0.01 between WT and RE or KO strains.

The degree of cell killing caused by a set of well established environmental stress challenges (H_2_O_2_, heat-shock and saline exposure) was analyzed in exponential-phase blastoconodia obtained from the wild type strain *C. parapsilosis* (AM2001/0013), its congenic *CpATC1* null mutant (KO) and the reintegrant strain (RE) containing a functional *CpATC1* gene. As shown in Figure 6, WT cells were the most sensitive under all the experimental conditions assayed, whereas the reintegrant strain (RE) cells showed an intermediate phenotype, although closer to parental than to *Cpatc1Δ* cells. In the case of the oxidative treatments, the addition of 5 mM H_2_O_2_ had only a limited effect on the viability of YPD-grown exponential phase cultures from the strains tested, while a higher concentration (50 mM H_2_O_2_) caused a more drastic loss of viability, the KO cells being significantly more resistant to oxidative stress (Fig. 6). Similar results were obtained when identical exponential-phase cultures were subjected to saline exposure (1.2 M NaCl). In contrast, a heat shock at 42°C caused a reduction of viability in the parental strain and, to a lesser extent, in RE cells, while the KO cells exhibited a greater capacity to withstand this moderate temperature (Figure 6). The results obtained with the heterozygous mutant (HET) were roughly equivalent to those found in the RE strain (results not shown).

### Trehalose content and trehalase activities in response to stress treatments

Identical cultures subjected to several stress conditions were used to evaluate the intracellular content of the protective disaccharide trehalose and the changes in the enzymatic activities involved in trehalose metabolism. The trehalose content showed significant differences between the control samples and stress-treated cells in the two strains analyzed, except for one saline treatment (Table 2). The intracellular trehalose levels increased in response to heat-shock in both strains, especially in the homozygous mutant (Table 2). Regarding the enzymatic activities, a significant activation of neutral trehalase was recorded in response to a thermal stress (42°C) in both strains, the increase being more pronounced in the parental strain. In turn, the acid trehalase activity did not change in response to the different stresses applied. As expected, *Cp*Atc1 activity was virtually undetectable in the KO mutant (Table 3).

**Table 2:** Intracellular content of trehalose following different stress treatments in exponential phase cultures of the parental strain (WT) and its congenic mutant deficient in acid trehalase (KO).

Treatment	Trehalose (nmol (mg wet wt) ^−1^)	
	WT	KO
Control	4.8±0.5	3.3±0.2
5 mM H_2_O_2_	8.3±0.2 ***	5.9±0.2***
50 mM H_2_O_2_	9.6±0.3***	6.2±0.1***
NaCl 1.2 M	3.7±0.5	4.1±0.1**
42°C	21.6±0.8***	24.2±0.6***

Yeast cells were grown at 30°C in YPD until they reached exponential phase (OD_600_  =  1.0–1.2). The samples were prepared and the trehalose content was measured as described in Methods. The results are the mean ± SD of one representative experiment of two performed in triplicate. The distinction between the treated samples and control values obtained was significant at **P<0.01 and ***P<0.001 according to the Student t-test.

**Table 3:** Levels of enzymatic activities corresponding to neutral (*Cp*Ntc1) and acid (*Cp*Atc1) trehalases in exponential phase cultures of the strain WT and *Cpatc1Δ* null mutant (KO) submitted to different stress treatments.

Treatment	Neutral Trehalase (*Cp*Ntc1) a		Acid trehalase (*Cp*Atc1) a	
	WT	KO	WT	KO
Control	16.6±0.8	14.2±0.4	3.1±0.3	<0.3
5 mM H_2_O_2_	17.1±0.3	12.1±1	3.7±0.4	<0.3
50 mM H_2_O_2_	12.4±0.3**	9.3±0.3***	2.9±0.2	<0.3
NaCl 1.2 M	20.5±1**	15.6±0.2**	3.5±0.3	<0.3
42°C	33.7±0.4***	22.5±0.5***	3.8±0.3	<0.3

The samples were prepared and the enzymatic activities were measured as described in Methods. The results are the mean ± SD of one representative experiment of two performed in triplicate. The distinction between the treated samples and control values obtained was significant at **P<0.01 and ***P<0.001 according to the Student t-test.

a Values are nmol glucose min^−1^ (mg protein) ^−1^.

### Induction of antioxidant activities in response to stress

**Figure 7:**
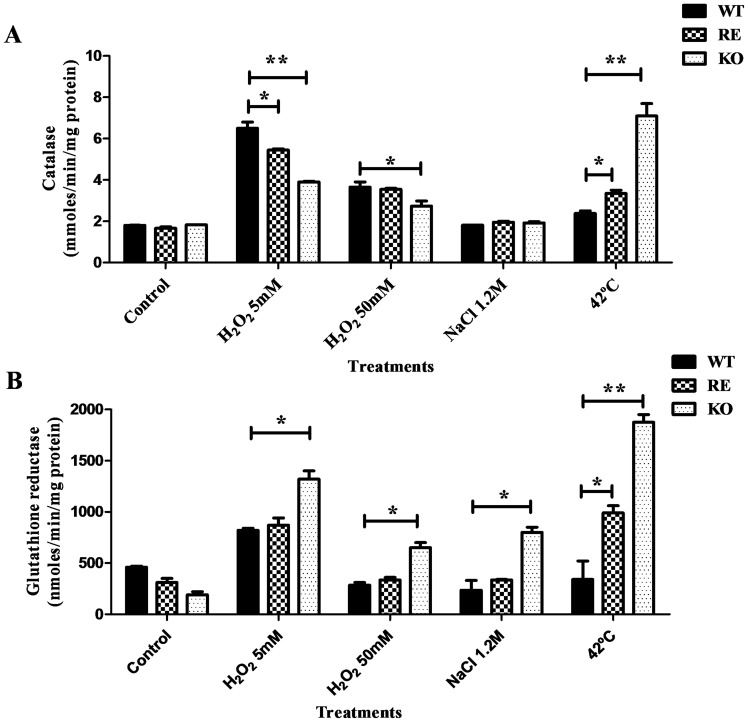
Effect of the exposure to different stress treatments on the enzymatic antioxidant responses in *C. parapsilosis*. The levels of catalase (**A**) and glutathione reductase (**B**) were determined in exponential phase cultures of the WT, KO and RE strains, submitted to different stress treatments. The samples were prepared and the enzymatic activities measured as described in Methods. Activity data are expressed with respect to an untreated control. The results are the mean ± SD of one representative experiment of two performed in triplicate. Student t-test: *P<0.05; **P<0.01 between WT and RE or KO strains.

Given that the ability of yeast to survive an acute oxidative stress depends on the induction of specific stress-responsive genes that encode for enzymes with both antioxidant and repairing roles, we analyzed the changes recorded in a set of activities that play an antioxidant role, in this case catalase and glutation reductase (GR) [30], [47]. For this purpose, exponential-phase cultures of the tested strains were exposed to identical stresses. Catalase activity levels varied depending on the treatment applied (Figure 7A). The basal activity increased after gentle oxidative stress (5 mM H_2_O_2_), but was only moderately activated in response to intense oxidative exposure (50 mM H_2_O_2_), while it remained at similar levels after saline/osmotic stress provoked by the addition of NaCl. This trend was observed in the parental, reintegrant and *CpATC1* null KO strains with no significant differences between them (Figure 7A). Note the high degree of catalase activation in KO cells (3-fold with respect to the control) when they were subjected to thermal stress (42°C), while the increase was significantly less conspicuous in the parental strain (Figure 7A). As regards glutation reductase, the results showed that this activity increased after mild or acute oxidative exposure (5 or 50 mM H_2_O_2_) in the acid trehalase-deficient mutant (Figure 7B). These data are consistent with the oxidative stress-induced GR activation observed in an *atc1Δ* mutant of *C. albicans* [17]. The application of NaCl to the cultures promoted a slight activation of the enzyme in parental cells, an effect that was much more pronounced and significantly higher in the homozygous mutant. Heating at 42°C was the most effective stress treatment for activating the GR activity in *Cp*Atc1-deficient cells, the RE cells showing a clear increase in such activity (Figure 7B).

### *CpATC1* is required for virulence in *C. parapsilosis*

**Figure 8:**
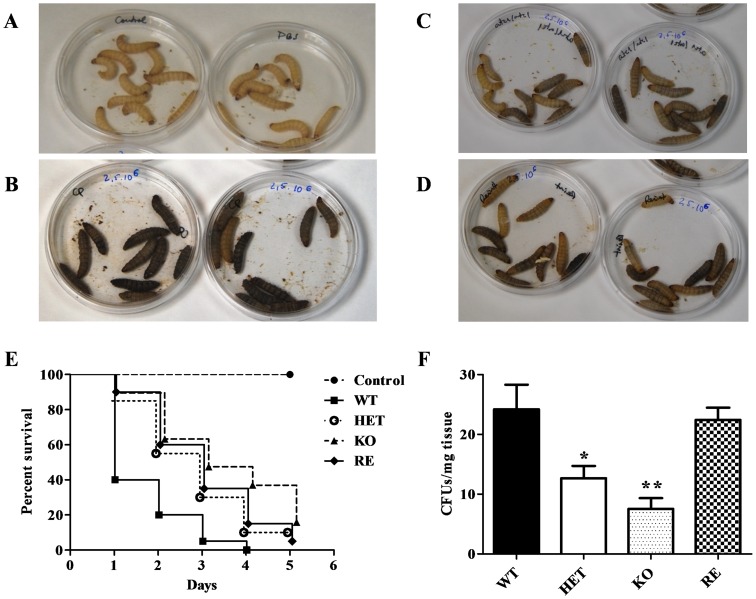
Virulence of *C. parapsilosis*. The degree of melanization in response to fungal infection was monitored by visual inspection of the *G. mellonella* larvae in control (**A**), WT strain (**B**), KO strain (**C**) and the reintegrant (RE) *C. parapsilosis* strain (**D**). Survival of *G. mellonella* larvae infected with 2.5×10^6^ yeast cells from each strain in PBS was monitored over time (**E**). The ability of *C. parapsilosis* to invade tissues was evaluated in a mouse model after intraperitoneal infection of Swiss mice (**F**). After 3 days of infection, the wild type (WT), heterozygous (HET), homozygous (KO) and reintegrant (RE) yeast strains were recovered from the kidneys and the CFUs/mg tissue were calculated. Ten mice were used per group and the experiment was repeated twice with similar results. Student t-test: *P<0.05; **P<0.01 between WT and HET, KO or RE strains.

The effect of deleting *CpATC1* on the virulence of *C. parapsilosis* was determined by using the larvae of the insect *Galleria mellonella*, which has proven to be a good model for candidiasis studies [48]. We first confirmed that the *C. parapsilosis* strains used in this study were capable of infecting and killing *G. mellonella* larvae (Figure 8 A-D). Following injection of WT cells, survival was reduced to approximately 40% within one day, compared with a 100% survival in the case of uninfected larvae, whereas the virulence was significantly attenuated through double deletion of *CpATC1* gene, and the heterozygous mutant showed an intermediate degree of infectivity (Figure 8 E). The virulence phenotype could be partially recovered by reintroduction of the native *CpATC1* gene (RE strain, Figure 8 E). It should be noted that *G. mellonella* larvae were melanized within a few minutes of *C. parapsilosis* WT strain injection, while such melanization was largely reduced in KO cells. Again, the RE strain showed an intermediate level of melanization (Figure 8 A-D). An important line of defense against fungal infections is the response shown by phagocytic cells [32]. For this reason, we studied whether WT and KO cells were recognized and phagocytosed by larval haemocytes to a similar extent in order to evaluate the degree to which they were responsible for the differences found in the virulence assays. Our results showed that there were no differences in the capacity of *G. mellonella* to engulf yeast cells among the *C. parapsilosis* strains 4 hours after infection (result not shown). Therefore, the decreased virulence in *CpATC1*-deficient cells compared to WT cells is probably not due to a diminished phagocytosis rate, but to the absence of a functional acid trehalase activity, that would act as a virulence factor. Similar results were obtained after analysis of the invasiveness in a mouse model, in which *C. parapsilosis* infection is not lethal, by intraperitoneal inoculation of the four strains under study in standard Swiss mice (Figure 8 F). The homozygous mutant in acid trehalase (KO strain) underwent a significant loss of virulence, as shown by the lower number of CFUs found in kidneys compared to those recorded in the WT strain three days after infection (Figure 8 F). In turn, the presence of a single copy of *CpATC1* gene (HET strain), as well as the reintroduction of the functional gene (RE strain), increased the invasiveness capacity as shown by the higher number of CFUs recovered (Figure 8 F).

## Discussion

*C. parapsilosis* is the causative agent in a high number, and increasing proportion, of invasive candidal infections [5]. Hence, it is of utmost importance to understand the molecular basis of *C. parapsilosis* virulence to be able to successfully combat this pathogen. In this work, we focus on the enzymes involved in the trehalose metabolism of *C. parapsilosis*, because trehalose has became a target of great interest in the search for novel effective antifungal compounds [13], [16], [17]. This sugar is absent in mammal cells, whereas trehalase is located in the brush border membranes of epithelial cells and in the kidney proximal tube [49]. A previous work showed that the *ATC1* gene, which codes for a cell-wall linked acid trehalase, is a virulence factor in *C. albicans*. Therefore, it also seemed conceivable that proteins located in the external surface might be preferential targets for antifungal drugs [17]. For this reason, we have carried out the cloning and functional characterization of an *ATC1* orthologous in *C. parapsilosis*.

An ORF (*CPAR2-208980*) was identified on contig 005809 by screening the data base of *C. parapsilosis*. Since the corresponding prospective protein had high homology with Atc1 of *C. albicans,* we called it *Cp*Atc1. The deduced amino acid sequence reveals the presence of a signal peptide at the N-terminus of the protein, which is a characteristic of proteins that transit through the secretory pathway. The theoretical molecular mass of mature *Cp*Atc1 protein is 116587.90 and it has 16 potential N-glycosylation sites (Figure 2A). These data are consistent with those described for other filamentous fungi and yeasts, e.g. *Emericella nidulans* and *C. albicans* [13], where the Atc1 activity is also located on the cell surface, in contrast to the acid trehalase from *S. cerevisiae* and *C. utilis*, which is located inside the vacuoles. This difference might reflect the existence of different exogenous trehalose uptake mechanisms in yeasts [13], [17], [50]–[52]. In filamentous fungi, trehalose hydrolysis appears to be carried out by an extracellular enzyme, while glucose is released after hydrolytic cleavage and then transported to the cell cytosol [52].

The homology model generated for the *Cp*Atc1 enzyme of *C. parapsilosis* revealed that the catalytic domain matches the catalytic domain of the glycosyl hydrolase family 65 (Figure 2A). In addition, it possesses two crucial catalytic residues, Glu570 and Asp442, present in other trehalases from *E. nidulans*, *S. cerevisiae* and *C. albicans* [13], which also overlap perfectly with the conserved residues of LbMP protein (Figure 3A).

The inability of the *Cpatc1Δ* null mutant to metabolize extracellular trehalose strongly supports the view that acid trehalase activity is necessary for trehalose hydrolysis in *C. parapsilosis*. These result are similar to those previously obtained for *C. albicans* [13], as well as for other fungi, such as *S. cerevisiae* [34] and *E. nidulans* [53]. When a phenotypic analysis of the *CpATC1* null mutant (KO) was performed to study its putative role in stress resistance and virulence, KO cells showed a greater capacity to withstand oxidative, osmotic and thermal challenges than those of its parental strain (Figure 6). These results are also consistent with those described for *C. albicans*, where an *atc1Δ* null mutant showed increased resistance to oxidative stress, heat and saline shock [17]. In *S. cerevisiae* too, the *ATH1* null mutant showed higher resistance to dehydration, freezing or ethanol-induced stress [54]. As regards endogenous trehalose, incubation at higher temperatures promoted the intracellular increase of this sugar (Table 2). *C. parapsilosis* displayed similar behavior during the heat shock response as *C. albicans* [17]. On the other hand, the two antioxidant enzymes monitored, catalase and GR, were activated in response to the type of stress applied (Figure 7). Thus, they might act as cell protectors as occurs in *C. albicans* [30]. The available data suggest that *Cp*Atc1 is a secretion protein that contains one signaling peptide and sixteen potential N-glycosylation sites (Figure 2), but they do not allow its location in the cell wall to be unequivocally established. In light of this, the observed stress resistance in KO cells could be due in part to structural modifications associated with *CpATC1* disruption, as the endogenous trehalose content accumulated in mutant and parental cultures was roughly equivalent (Table 2).

Since double disruption of the *CpATC1* gene seems to alter the stability of the external surface, leading to a reduction in virulence, we next studied the impact of *CpATC1* on virulence. The innate immune responses of mammals are involved in the defense against fungal pathogens [55]. Since components of the innate immune response are conserved between mammals and insects, analysis of insect responses to fungal pathogens can provide general insights into the process of host defense against fungi [56]–[58]. In recent years, there has been much interest in developing non-mammalian host models to study microbial infectivity in order to attenuate the bioethical impact of classical animal experimentation. In this respect, *Galleria mellonella* is a Lepidoptera that has been successfully used as a model host to study the virulence of pathogenic fungi, such as *Cryptococcus neofomans*, [59] *C. albicans* [60] and *Aspergillus fumigatus* [61]. Our results show that the validity of this model can be extended to *C. parapsilosis.*

The findings strongly support the idea that the genes coding for an acid trehalase (*ATH1/ATC1*) involved in trehalose catabolism are necessary for both virulence and resistance to environmental stress exposure in several pathogenic yeast species [17]. Therefore, the corresponding enzymatic moiety deserves more intensive research as a potential target for the development of new, more potent and specific antimycotic drugs.
